# Type 3 macular neovascularization in a patient with pentosan polysulfate maculopathy

**DOI:** 10.1016/j.ajoc.2022.101771

**Published:** 2022-12-05

**Authors:** Elodie Bousquet, Brian A. Lee, Ahmad Santina, SriniVas Sadda, David Sarraf

**Affiliations:** a Retinal Disorders and Ophthalmic Genetics Division, Stein Eye Institute University of California Los Angeles, David Geffen School of Medicine at UCLA, Los Angeles, CA, United States; b Doheny Eye Institute, Department of Ophthalmology, University of California Los Angeles, Los Angeles, CA, United States; c Greater Los Angeles VA Healthcare Center, Los Angeles, CA, United States

**Keywords:** Anti-VEGF, Multimodal imaging, Pentosan polysulfate sodium maculopathy, Type 3 macular neovascularization

## Abstract

**Purpose:**

To report the development of type 3 macular neovascularization (MNV) in a patient with pentosan polysulfate sodium (PPS) maculopathy one year after PPS cessation.

**Observation:**

A 72-year-old woman presented for decreased visual acuity in the left eye. Medical history was significant for interstitial cystitis treated with PPS for 11 years (cumulative dose of 1205 g) and PPS maculopathy. PPS was discontinued 1 year prior to presentation. Blue-light fundus autofluorescence and spectral domain optical coherence tomography confirmed the diagnosis of bilateral PPS maculopathy. OCT-angiography illustrated the development of type 3 MNV with intraretinal fluid in the left eye. Intravitreal injections of aflibercept were initiated with a good visual and anatomical response.

**Conclusion and importance:**

This report describes the development of type 3 MNV in a patient with PPS macular toxicity one year after PPS cessation. This complication emphasizes the need for regular retinal surveillance even after discontinuation of the inciting drug.

## 1 Introduction

Pentosan polysulfate sodium (PPS) is an oral treatment for interstitial cystitis, a debilitating disease with few other effective therapies. Long term PPS exposure is associated with a progressive, vision-threatening pigmentary maculopathy first described in 2018 by Pearce et al.^1^ PPS maculopathy is typically characterized by a speckled hyper and hypo-autofluorescent pattern centered on the fovea, and around the optic disc in more advanced cases.^1^^,^^2^ The prognosis of this condition is variable but the maculopathy is not reversible^3^ and may progress despite PPS discontinuation.^4^ Type 1 macular neovascularization (MNV), originating from the choroid, can complicate this condition.^5^, ^6^, ^7^

Type 3 MNV, previously known as retinal angiomatous proliferation (RAP), refers to intraretinal neovascularization extending from the deep retinal capillary plexus into the outer retina and retinal pigment epithelium.^8^, ^9^, ^10^, ^11^ Type 3 MNV occurs almost invariably in eyes with age-related macular degeneration (AMD), but can very rarely complicate other conditions such as retinitis pigmentosa and enhanced S-cone syndrome.^12^^,^^13^

In this report, we present a case of type 3 MNV that developed in a patient with PPS maculopathy one year after PPS cessation.

## 2 Case report

A 72-year-old woman presented acutely with blurred vision in the left eye. Medical history was remarkable for interstitial cystitis treated with PPS for 11 years, with a cumulative dose of 1205 g. Ocular history was significant for PPS maculopathy and the PPS treatment was discontinued one year prior to presentation.

On examination, best-corrected visual acuity (BCVA) was 20/25 in the right eye and 20/60 in the left eye. The anterior segment examination was unremarkable in both eyes. Retinal examination and color fundus photography revealed macular pigmentary degeneration in both eyes. Fundus autofluorescence (FAF) showed a speckled pattern of hypo and hyper-autofluorescence around the fovea and the optic disc, extending toward the temporal arcades in both eyes without evidence of retinal pigment epithelium (RPE) atrophy and consistent with grade 1 PPS maculopathy.^2^^,^^7^

**Fig. 1:**
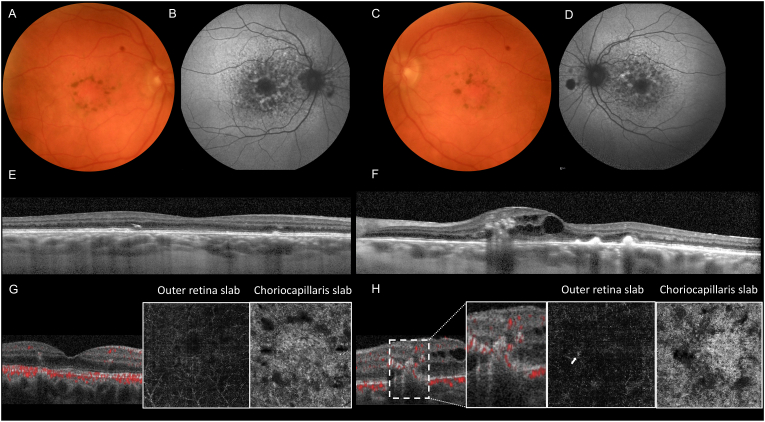
**Multimodal imaging of 72-year-old with pentosan polysulfate sodium maculopathy associated with type 3 macular neovascularization (MNV) in the left eye. (A and C)** Fundus photography shows pigmented lesions in the macula OU (**B and D)** Fundus autofluorescence (FAF) illustrates a speckled pattern of hypo and hyper-autofluorescence around the fovea and the optic disc, extending toward the temporal arcades OU. (**E and F)** Spectral domain-optical coherence tomography (SD-OCT) illustrates areas of ellipsoid zone and retinal pigment epithelium disruption OU. SD-OCT OS **(F)** shows intraretinal fluid associated with outer retinal hyperreflective foci (HRF) suspicious for type 3 MNV. (**G and H)** OCT-angiography (OCT-A) (performed 15 days after the type 3 MNV diagnosis) shows no abnormal flow in the right eye (G) but abnormal flow corresponding to the HRF on both the cross sectional OCT overlay and the en face OCT-A (arrow), confirming type 3 MNV in the left eye. Choriocapillaris slabs show prominent flow deficits (in black) in both eyes suggestive of inner choroidal ischemia, although some of these may be explained by shadow artifact OU.

Spectral domain-OCT (SD-OCT) (Heidelberg Engineering, Heidelberg, Germany) illustrated areas of ellipsoid zone and RPE disruption in both eyes. OCT of the left eye also showed intraretinal fluid and hyperreflective foci (HRF) suggestive of type 3 MNV. OCT-Angiography (OCT-A) (Optovue, Fremont, California, USA) confirmed the presence of flow within the intraretinal hyperreflective lesion descending towards the outer retina consistent with type 3 MNV OS. OCTA with segmentation of the choriocapillaris showed prominent flow deficits (in black) in both eyes, suggestive of underlying choriocapillaris ischemia, although some of these deficits may be explained by overlying blocking defects in the outer retina. (Fig. 1).

**Fig. 2:**
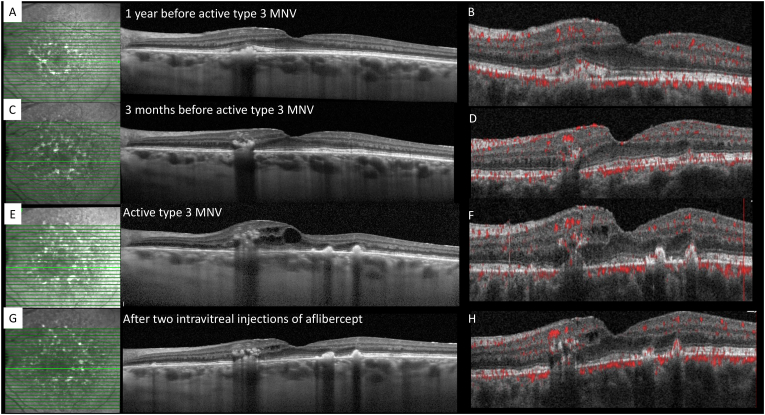
**Spectral domain-optical coherence tomography (SD-OCT) and OCT-angiography (OCT-A) show the progressive development of type 3 macular neovascularization (MNV)and the response to intravitreal injection of aflibercept**. **A and B:** SD-OCT and OCT-A at 1 year prior to baseline presentation. **C and D:** SD-OCT and OCT-A at 3 months prior to baseline presentation. **E and F:** SD-OCT and OCT-A at baseline presentation. **G and H:** SD-OCT and OCT-A after 2 intravitreal injections of aflibercept. **A and B:** SD-OCT **(A)** shows outer retinal hyperreflective foci (HRF) at the site of subsequent development of type 3 MNV but OCT-A **(B)** fails to show evidence of corresponding flow. **C and D:** SD-OCT **(C)** shows HRF higher in the retina in the outer nuclear layer, outer plexiform layer and inner nuclear layer and OCT-A **(D)** shows flow corresponding to the HRF. **E and F:** SD-OCT **(E)** and OCT-A **(F)** show active type 3 MNV associated with intraretinal fluid. **G and H:** One month after 2 monthly intravitreal injections of aflibercept, a significant reduction of fluid is observed with SD-OCT **(G)**, although persistent flow is noted corresponding to the type 3 MNV with OCT-A **(H)**. Visual acuity improved from 20/60 to 20/30 OS.

SD-OCT and OCT-A performed at one year and at three months before the development of active type 3 MNV are both shown in Fig. 2. One year before the detection of active type 3 MNV, outer retinal HRF were observed, contiguous with the RPE, at the site of subsequent type 3 MNV. Three months before the detection of active type 3 MNV, the HRF were identified at the level of the outer nuclear, outer plexiform and inner nuclear layers, at which time flow was noted within the HRF on OCT-A.

The patient was treated with 2 monthly intravitreal injections of aflibercept in the left eye. Intraretinal fluid nearly resolved (Fig. 2) and visual acuity improved from 20/60 to 20/30 OS.

## 3 Discussion

This report illustrates a case of type 3 MNV developing in a patient with PPS maculopathy one year after PPS cessation. To our knowledge, this report is the first to describe type 3 MNV as a complication of PPS maculopathy. While this patient exhibited classic features of PPS maculopathy in each eye, there was a notable absence of AMD findings such as macular drusen. Cystoid macular edema, acquired vitelliform lesions and type 1 and 2 MNV have all been reported as complications of PPS maculopathy.^2^^,^^3^

Recent progress in retinal imaging with OCT-angiography and histologic analyses have confirmed a deep retinal capillary plexus origin of type 3 MNV in AMD.^9^, ^10^, ^11^^,^^14^

In this case of type 3 MNV secondary to PPS maculopathy, HRF were detected in the ONL and OPL three months before the onset of active type 3 MNV. Using OCT-A, flow was detected inside the HRF, which may represent early intraretinal vascular proliferation originating from the DCP, referred to as “nascent type 3 MNV” by Sacconi et al.^15^

The development of T3 MNV may be the result of choroidal ischemia. Using OCT-A, multiple studies have shown significant impairment of flow (or perfusion) within the inner choroid or choriocapillaris of eyes with type 3 MNV that may even exceed the severity of choroidal ischemia in eyes with type 1 MNV.^16^^,^^17^ It has been speculated that a severely impaired choriocapillaris may be less capable of driving the development of type 1 MNV, which may encourage ischemic RPE cells to migrate into the retina (evident as HRF) and stimulate an angiogenic response from the DCP in the form of type 3 MNV.^18^ Of interest, Wang et al.^7^ showed evidence of choroidal thinning with OCT in eyes with PPS maculopathy and Fogel-Levin et al.^19^ illustrated significant choriocapillaris non perfusion with OCT-A in eyes exposed to PPS but without retinal toxicity indicating that inner choroidal impairment may be the earliest evidence of PPS related ocular toxicity. The growth of type 3 MNV in this case of PPS maculopathy may be driven therefore by inner choroidal ischemia.

In conclusion, this report describes the presence of type 3 MNV in a patient with PPS maculopathy one year after PPS discontinuation. Significant improvement of intraretinal fluid and visual acuity was noted after anti-VEGF therapy. Pentosan polysulfate sodium may be primarily toxic to the choroid and inner choroidal ischemia may explain the subsequent development of MNV, including type 3 neovascularization. This case emphasizes the importance of close monitoring of patients with PPS maculopathy for neovascular and exudative complications even after PPS cessation.

## Patient consent

Signed consent to use medical data for research purposes was obtained from the patient.

## Acknowledgments and disclosures

No funding or grant support

## Declaration of competing interest

Authors with financial interests or relationships to disclose are listed below.

Dr Elodie Bousquet receives personal fees from Allergan and Bayer; Dr. Srinivas Sadda receives personal fees from Allergan/Abbvie, Amgen, Apellis, Iveric, Oxurion, Novartis, Bayer, Regeneron, Roche/Genentech, Nanoscope, Notal, Boerhinger Ingelheim, Biogen, Optos, Centervue, Heidelberg Engineering, and Nidek; research grants from 10.13039/501100002806Carl Zeiss Meditec, and non-financial support from Optos, Centervue, 10.13039/501100010313Heidelberg Engineering, 10.13039/501100010383Topcon, Carl Zeiss Meditect, and Nidek.

Dr David Sarraf receives grants, personal fees from Amgen, personal fees from Bayer, grants from Boehringer, grants from 10.13039/100004328Genentech, non-financial support from Heidelberg, personal fees from Novartis, personal fees, non-financial support from Optovue, grants from 10.13039/100009857Regeneron, non-financial support from 10.13039/501100010383Topcon, personal fees from Iveric Bio.

The following authors have no financial disclosures: BL, AS.

All authors attest that they meet the current ICMJE criteria for Authorship.
